# A definition for community-based surveillance and a way forward: results of the WHO global technical meeting, France, 26 to 28 June 2018

**DOI:** 10.2807/1560-7917.ES.2019.24.2.1800681

**Published:** 2019-01-10

**Authors:** Technical Contributors to the June 2018 WHO meeting

**Affiliations:** 1 Technical Contributors to the June 2018 WHO meeting are listed at the end of the article

**Keywords:** public health surveillance, epidemiological surveillance, community-based surveillance, community health volunteers, community health workers, community participation

Public health surveillance is used for the early detection of public health events and the monitoring of the health status of a population to guide and assess the impact of interventions [1].

In most settings, public health surveillance relies on information collected by healthcare facilities when patients come to seek treatment. This approach is limited in its ability to detect public health events and the occurrence of disease in populations that do not seek treatment at healthcare facilities or that experience barriers to treatment. This may be the case, for example, in hard-to-reach areas, areas where the population relies highly on traditional healers or alternative treatments, or in populations with stigmatising illnesses such as HIV infections. Engaging community members to collect health information from within their communities and report it for public health surveillance purposes is increasingly gaining interest as an approach to address such limitations. This approach is conventionally termed ‘community-based surveillance’ (CBS).

Surveillance strategies such as the Integrated Disease Surveillance and Response technical guidelines of the World Health Organization (WHO) Regional Office for Africa [2] acknowledge the role of community members in reporting cases of epidemic-prone diseases and unusual health events. CBS has been used in disease eradication programs including smallpox, guinea worm and polio [3], as well as during the West African Ebola virus disease outbreak of 2014–15, where community health workers and volunteers played a role in early detection and timely reporting to the health system [4].

To better understand the concept of and procedures for CBS, the Laboratory and Surveillance Strengthening team in the WHO Health Emergencies Programme undertook a systematic literature review. This review identified a lack of consensus on the terms and definitions available for CBS, along with a wide diversity in the characteristics of past and existing CBS.

These results highlighted the need for a meeting to convene country and partner representatives with experience in CBS implementation to: (i) collectively define the term ‘community-based surveillance’, (ii) identify good practices and challenges for CBS implementation and operation, and (iii) identify priority activities to support countries in implementing and strengthening CBS. In an effort to achieve these objectives, WHO organised a technical meeting on CBS on 26–28 June 2018 at the WHO office in Lyon, France. The meeting brought together 28 participants from several countries (Cameroon, Ghana, Mongolia, Sierra Leone, Thailand, UK) and partner organisations (Africa Centres for Disease Control and Prevention, CARE International, United States of America Centers for Disease Control and Prevention, CORE group, International Federation of Red Cross and Red Crescent Societies, International Rescue Committee, Norwegian Red Cross, World Vision International). A participatory methodology was used, with minimum plenary presentations; instead, the meeting consisted of working groups with various participatory methods (detailed agenda with full methodology available in Supplement S1).

## Definition of CBS

A consensus definition of CBS was adopted: ‘CBS is the systematic detection and reporting of events of public health significance within a community by community members.’

The following characteristics of strong CBS were provided with the definition: it should be integrated in a formal surveillance structure, be actionable and timely, and have perceived benefits to the community, well-defined reporting mechanisms, a feedback mechanism and a monitoring and evaluation process.

Extensive discussions were held on the wording of the definition, and it was determined that the term ‘community’ should be clarified in annex of the definition. The consensus was that the definition of a community cannot be restricted to a certain geographical area; while it may be difficult to produce an all-encompassing definition of community, it is important that each CBS clearly defines its community under surveillance.

In addition, the following language specifications were suggested: use of the term ‘systematic’ instead of ‘system’ to avoid the connotation of CBS being a separate vertical system, while also ensuring that CBS is defined as a structured, formal process; use of the term ‘events of public health significance’ to encompass not only unusual events, but also any condition, disease or event that has implications for public health; specification of detection and reporting ‘by community members’ to imply that if event detection and reporting is conducted by a person not from the community itself, it cannot be described as CBS. The latter was described as the most defining aspect of CBS. The term ‘community member’ was defined as any person belonging to the community under surveillance.

## Subgroups of CBS needing specific guidance

Participants selected three CBS subgroups for further discussion, based on perceived importance, from 18 that had been proposed: (i) CBS in the context of a non-functioning routine surveillance system, (ii) CBS for hard-to-reach populations and (iii) CBS in a community lacking social cohesion.

After a thorough discussion, participants concluded that—in addition to generic CBS guidance and tools, which would apply in most situations—specific guidance and tools were only required for CBS in the context of a non-functioning routine surveillance system.

For CBS in hard-to-reach populations and communities lacking social cohesion, no specific additional guidance was identified as needed; however, these special contexts need to be addressed in any generic guidance and tools supporting CBS.

## Good practices and challenges

Participants identified good practices in CBS implementation and operation (Box 1).

Box 1 **Good practices in community-based surveillance implementation and operation, WHO global technical meeting, France, 26–28 June 2018**

• Integration of CBS within the overall surveillance system, including involvement of different administrative levels (i.e. local, intermediate, central) in the CBS data reporting mechanism, so that each level can make use of the data (with the possible exception of emergency situations, where this might not be feasible);

• Adaptation of CBS to local needs and context, including identification of diseases and events that are of interest to the community, understanding the local perception of diseases and use of local terminology to describe diseases, signals or case definitions;

• Adaptation of tools and approaches for data collection, reporting and communication to suit the community;

• Reinforcement of community and government ownership of CBS to promote sustainability;

• Coordination between different partners (i.e. organisations working in the community) and sectors (e.g. human health, animal health, environmental health) to enhance data and knowledge sharing, harmonise tools and approaches for interoperability and prevent duplication of activities in the community;

• Selection of data collectors that are trusted and well-connected within the community and socio-culturally appropriate and inclusive (particularly with regard to gender);

• Development and utilisation of selection criteria for the recruitment of data collectors;

• Provision of regular training, supervision and appropriate incentives (i.e. valued by the community) to motivate data collectors;

• Simple and purposeful data collection process (i.e. simple signals, tools and methods), based on clearly defined objectives and priorities of CBS;

• Simple mechanism to analyse data are in place before CBS becomes operational;

• Provision of observable benefits to the community (e.g. interventions, health education);

• Establishment of a mechanism for information feedback and communication with the community.

CBS: community-based surveillance; WHO: World Health Organization.

Participants also identified challenges in CBS implementation and operation, some of which overlapped with the good practices (Box 2).

Box 2 **Challenges in community-based surveillance implementation and operation, WHO global technical meeting, France, 26–28 June 2018**

• Accessing hard-to-reach populations, including those that are hidden because of stigmatisation, oppression or being otherwise neglected;

• Selecting suitable community members for data collection and reporting: difficulties to follow the selection criteria for recruitment in case of pressure from influential community members;

• Overburdening of data collectors, who are often given multiple responsibilities and may be involved in multiple CBS and community-level programs;

• Waning interest and motivation of data collectors over time, which often leads to deteriorating quality in reporting and high turnover of staff;

• Providing incentives to motivate data collectors;

• Balancing the sensitivity and specificity of CBS , as simple and broad signal definitions make CBS more sensitive but less specific;

• Sustainability of CBS;

• Existence of parallel surveillance systems within a community can create duplication of work and required resources, with limited or no interoperability of the data and processes;

• Logistics management and operational costs, in particular the use of digital technologies;

• Providing regular training, supervision and feedback to data collectors;

• Providing observable benefits to the community;

• Coordinating with partner organisations and government.

CBS: community-based surveillance; WHO: World Health Organization.

## Needs and gaps to replicate good practices and address challenges

Participants were presented with the existing CBS guidance and tools retrieved during a systematic literature review [4-9].

Available guidance and recommendations for data collectors were deemed more or less sufficient, though needing some updates, whereas many gaps were identified for the other aspects. While the existing guidance and tools address some of the identified needs, they are scattered across several documents, highlighting the need to consolidate them into one single guide or knowledge repository.

Participants expressed that any guidance or tool for CBS needs to be based on field experience that has been evaluated for effectiveness or based on appropriate research in different settings; further, it should be accompanied by case studies of good practices or illustrated with examples.

## Activities to support CBS implementation and operation: the way forward

Based on the gaps identified as obstacles to replicating good practices and addressing challenges, participants selected 11 priority activities to support CBS implementation and operation, and ranked them through a scoring method [10] (methodology described in Supplement S1). These priority activities and the number of points they received are shown in Box 3.

Box 3 **Priority activities to support community-based surveillance implementation and operation, ranked by score, WHO global technical meeting, France, 26–28 June 2018**

• Develop and compile case studies of existing CBS, covering the lessons learnt, challenges and significant aspects of CBS (e.g. implementation, setting, purpose, actors, data collection and reporting, feedback and communication, monitoring and evaluation, community involvement, effectiveness, sustainability, costs). (143 points)

• Develop global CBS guidelines that bring together all of the existing guidance and tools, which are currently scattered over different documents, and fill existing gaps including guidance for hard-to-reach populations and communities lacking social cohesion. (114 points)

• Create a CBS community of practice with a repository of available material to act as an exchange channel for experts from different levels (e.g. regional, national) and for different areas (e.g. emergency setting, cross-border, mobile population). (111 points)

• Conduct a systematic literature review and additional research on incentives and motivating factors for CBS. (107 points)

• Develop a CBS resource library. (102 points)

• Develop CBS guidance to address hard-to-reach populations. (101 points)

• Conduct workshops on how to design a context-specific CBS. (97 points)

• Develop standard operating procedures for selection and training of CBS data collectors and supervisors. (83 points)

• Conduct research on One Health approach in CBS. (77 points)

• Develop communication packages to advocate for CBS. (75 points)

• Update the 2001 guide for CBS published by the Academy for Educational Development [5], including a section on protection from stigma. (51 points)

CBS: community-based surveillance; WHO: World Health Organization.Meeting participants selected 11 priority activities to support CBS implementation and operation, and ranked them through a scoring method [10].

## Conclusion

The meeting achieved its expected outcomes by providing a consensus definition of CBS, a list of good practices and challenges for CBS, and a ranked list of priority activities to strengthen CBS. Participants expressed both willingness and motivation to contribute to these activities. Moving forward, they requested that WHO take a coordinating and facilitating role in the development of global standards of practices.

## Notes

**Technical Contributors to the June 2018 WHO meeting:** José Guerra, World Health Organization (WHO), Lyon, France; Yolanda Bayugo, World Health Organization (WHO), Lyon, France; Pratikshya Acharya, World Health Organization (WHO), Lyon, France; Michael Adjabeng, Ghana Health Service, Accra, Ghana; Céline Barnadas, World Health Organization (WHO), Lyon, France; Saverio Bellizzi, World Health Organization (WHO) Regional Office for the Eastern Mediterranean, Cairo, Egypt; Simon Cottrell, Public Health Wales, Cardiff, United Kingdom; Dora Ward Curry, CARE-US, Atlanta, United States of America; Yannick Kamga, Ministry of Public Health, Yaoundé, Cameroon; Barbara Jones, International Rescue Committee, Monrovia, Liberia; Anine Kongelf, Norwegian Red Cross, Oslo, Norway; Katrina Litam, World Health Organization (WHO), Lyon, France; Sharifa Merali, Synergy America, Inc., contractor to National Center for Immunization and Respiratory Diseases, Centers for Disease Control and Prevention, Atlanta, United States of America; Masami Miyakawa, World Health Organization (WHO) Regional Office for the Western Pacific, Manila, Philippines; Roxanne Moore, International Federation of Red Cross and Red Crescent Societies, Vernier, Switzerland; Anthony W Mounts, Center for Global Health, United States Centers for Disease Control and Prevention, Hanoi, Viet Nam; Jonathan Polonsky, World Health Organization (WHO), Geneva, Switzerland; Jukka Pukkila, World Health Organization (WHO) Regional Office for Europe, Copenhagen, Denmark; Suvdmaa Nyam, Ministry of Health, Ulanbaatar, Mongolia; Leonard Peruski, Center for Global Health, Centers for Disease Control and Prevention, Atlanta, United States of America; Ando Tiana Raobelison, World Vision International, Nairobi, Kenya; Soatiana Rajatonirina, World Health Organization (WHO) Regional Office for Africa, Brazzaville, Congo; Thomas Takpau Samba, National Public Health Agency, Freetown, Sierra Leone; Roma Solomon, CORE Group Polio Project, Gurgaon, India; Patcharin Tantiworrawit, Ministry of Public Health, Bangkok, Thailand; Irène Yaméogo Ngendakumana, Eagle Global Services, contractor to United States of America Centers for Disease Control and Prevention, Ouagadougou, Burkina Faso; Ahmed Zaghloul, Africa Centres for Disease Control and Prevention, Addis Ababa, Ethiopia; Arunmozhi Balajee, National Center for Immunization and Respiratory Diseases, Centers for Disease Control and Prevention, Atlanta, United States of America; Sébastien Cognat, World Health Organization (WHO), Lyon, France.
